# Peripheral and intestinal mucosal-associated invariant T cells in premature infants with necrotizing enterocolitis

**DOI:** 10.3389/fphar.2022.1008080

**Published:** 2022-09-14

**Authors:** Jiayi Tian, Chaoying Yan, Yanfang Jiang, Haohan Zhou, Liyuan Li, Jingjing Shen, Jian Wang, Hongyu Sun, Guang Yang, Wei Sun

**Affiliations:** ^1^ Center for Reproductive Medicine and Center for Prenatal Diagnosis, First Hospital, Jilin University, Changchun, China; ^2^ Department of Neonatology, The First Hospital of Jilin University, Changchun, China; ^3^ Department of Center of Gene Diagnosis, The First Hospital of Jilin University, Changchun, China; ^4^ Department of Orthopaedic Oncology, Changzheng Hospital, Second Military Medical University, Shanghai, China; ^5^ Department of Molecular Biology, College of Basic Medical Sciences, Jilin University, Changchun, China; ^6^ School of Civil Engineering and Architecture, Taizhou University, Taizhou, China

**Keywords:** necrotizing enterocolitis, mucosal-associated invariant T cells, preterm, inflammatory cytokines, T cells

## Abstract

**Background:** Necrotizing enterocolitis (NEC) is a potentially fatal inflammatory gastrointestinal disease in preterm infants with unknown pathogenesis. Mucosal-associated invariant T (MAIT) cells primarily accumulate at sites where exposure to microbes is ubiquitous and regulate immunological responses. As the implications of these cells in NEC development in premature infants remain unknown, we investigated the role and characteristics of MAIT cells in NEC pathogenesis.

**Methods:** The percentage of different MAIT cell subsets in peripheral blood samples of 30 preterm infants with NEC and 22 control subjects was estimated using flow cytometry. The frequency of MAIT cells in the intestinal tissues of five NEC patients and five control subjects was also examined. The level of serum cytokines was estimated using cytometric bead array. Potential associations between the different measurements were analyzed using the Spearman’s correlation test.

**Results:** Compared with controls, the NEC patients were found to have significantly reduced percentages of circulating CD161^+^ CD3^+^ CD8αα^+^ T cells and CD161^+^ CD3^+^ TCRγδ^-^TCRVa7.2^+^ MAIT cells. In the intestinal tissues, the percentage of MAIT cells was significantly higher in samples from the NEC patients than the controls. Furthermore, the percentage of circulating MAIT cells in the peripheral blood samples was inversely correlated with that in the intestinal tissues of the NEC patients. The percentage of CD8αα^+^ MAIT cells was found to be significantly reduced in both peripheral blood and intestinal tissues of NEC patients. Following treatment, the frequency of circulating MAIT cells significantly increased in NEC patients and reached a level similar to that in the control subjects. However, there was no difference in the percentage of circulating CD8αα^+^ MAIT cells before and after treatment in the NEC patients.

**Conclusion:** Our results suggested that during the development of NEC MAIT cells accumulate in the inflammatory intestinal tissues, while the percentage of CD8aa^+^ MAIT cells is significantly decreased, which may lead to the dysfunction of MAIT cells in gut immunity.

## Introduction

Necrotizing enterocolitis (NEC) is a severe inflammatory gastrointestinal disease that commonly affects preterm infant, with high morbidity and mortality. It is characterized by ischemic necrosis of intestinal mucosa, and is associated with excessive intestinal inflammation and invasion of the immature gut by enteric gas-forming bacteria (Neu, 1996). Although the exact pathogenesis of NEC remains poorly understood, accumulating evidence is suggesting that the inflammation caused by abnormal bacterial colonization and the immaturity of the immune system in preterm infants are crucial for its development (Hunter et al., 2008; Nanthakumar et al., 2011; Tanner et al., 2015).

Recent studies on human and animal models have shown that innate immune cell responses are associated with the development of NEC (Weitkamp et al., 2014; Tanner et al., 2015; Wei and Besner, 2015; Egan et al., 2016). In the ileum of premature infants with NEC, a reduction in the number of lamina propria regulatory T cells has been reported to contribute to the disease progression (Weitkamp et al., 2013). Additionally, a study using a murine model of NEC showed that pro-inflammatory macrophages promoted NEC by increasing the rate of apoptosis in the intestinal epithelial cells (Wei and Besner, 2015). T cell receptor (TCR) γδ^+^ intraepithelial lymphocytes have been reported to migrate to and remain in the intestinal epithelium during early embryogenesis, and their loss is associated with the development of NEC (Weitkamp et al., 2014).

Mucosal-associated invariant T (MAIT) cells are innate-like T cells and have been identified as CD3^+^ TCRγδ^-^Vα7.2^+^ CD161^+^ T cells (Gapin and Check, 2014; Serriari et al., 2014; Ling et al., 2016). They are abundantly found in the peripheral blood, intestinal mucosa, and liver of humans (Treiner et al., 2005; Dusseaux et al., 2011). MAIT cells can be classified into CD8^+^, CD4^+^, and CD8^−^CD4^−^ (double-negative, DN) subsets. CD8^+^ MAIT cells, which regulate inflammatory immune responses, can be further classified into CD8αα and CD8αβ subsets (Le Bourhis et al., 2010; Walker et al., 2012). Activated MAIT cells secrete several cytokines essential for defense against pathogenic infections (Le Bourhis et al., 2010; Le Bourhis et al., 2013). MAIT cells have also been reported to participate in immune response against inflammatory gastrointestinal diseases such as the inflammatory bowel disease, a disorder characterized by dysregulated immunity (Serriari et al., 2014; Booth et al., 2015). However, the association between MAIT cells and NEC remains unknown.

Given the dysregulated intestinal immune response and imbalanced microbial flora in the gut during NEC, we hypothesized that MAIT cells might play a role in the pathogenesis of NEC. To test this hypothesis, we analyzed the distribution of the different subsets of MAIT cells in the peripheral blood and intestinal tissue samples from premature infants with NEC and compared them with those in control subjects using flow cytometry. We also analyzed the clinical relevance of MAIT cells in NEC patients by estimating the frequencies of the different subsets of MAIT cells before and after treatment.

## Materials and methods

### Subjects and samples

All participants were recruited from the Department of Neonatology, the First Hospital, Jilin University, Changchun, China. The experimental protocol used was approved by the Human Ethics Committee of Jilin University. Written informed consent was obtained from the parents of each subject. A total of 30 patients diagnosed with NEC at stage II (*n* = 22) or advanced NEC at stage III (*n* = 8) were recruited in this study. The staging was performed according to the modified Bell’s staging criteria (Walsh and Kliegman, 1986). During the same period, 22 other gender- and gestational age-matched infants were recruited as control subjects. These infants showed no evidence of NEC or infection, including respiratory distress, abdominal distension, feeding intolerance, lethargy, irritability, or temperature instability (Benkoe et al., 2013). All the infants chosen were born premature (<37 weeks of gestation). Peripheral blood samples were collected from the 30 NEC patients and the 22 control subjects. Surgically resected intestinal tissue samples were obtained from 5 of the 8 stage III NEC patients and 5 of the 22 control subjects, who had undergone surgical treatment for intestinal atresia (*n* = 3) and intestinal malrotation (*n* = 2). All the participants were subjected to routine laboratory tests for estimating full blood cell counts and concentration of serum C-reactive proteins (CRP). The demographic and clinical characteristics of the subjects are shown in Table 1.

**TABLE 1:** Demographic and clinical characteristics of study participants.

Parameters	CONTROL		NEC	
	Blood (*n* = 22)	Tissue (*n* = 5)	Blood (*n* = 30)	Tissue (*n* = 5)
Male/Female (n)	9/13	2/3	13/17	2/3
Birth weight (g)	1870 (1150–3650)	1650 (1200–2560)	1740 (1100–3570)	1300 (1100–2470)
Gestational age (weeks)	32 (29–36)	31 (29–34)	32 (27–36)	29 (27–31)
White blood cells (10^9^/L)	9.9 (5.78–23.5)	11.2 (9.8–15.4)	8.06 (2.82–20.26)	8.96 (6.32–18.75)
Lymphocytes (10^9^/L)	3.75 (1.76–7.63)	3.11 (1.94–4.29)	3.18 (0.62–9.55)	3.63 (2.68–9.55)
CRP (mg/L)	1.42 (0.40–3.90)	0.92 (0.68–3.33)	11.07 (0.67–122)*	7.7 (0.67–47.8)*

Data are expressed as the median (range or case number). *p < 0.05 vs. the control.

### Treatment and follow-up

The strategy for managing NEC depends on the severity of illness as classified by the Bell’s staging criteria (Walsh and Kliegman, 1986). Medical management should include supportive care, antibiotic therapy (Solomkin et al., 2010), and close laboratory and radiologic monitoring. Surgical intervention was performed in the patients with advanced NEC and bowel perforation (stage III B). The patients were followed up for 2–3 weeks. There were six stage-II NEC patients with complete records, while the other 24 patients failed to follow-up. Blood samples were collected before and 2–3 weeks after treatment.

### Isolation of peripheral blood mononuclear cells

PBMCs were extracted from 0.5 ml of heparinized blood using density gradient centrifugation with Ficoll-Paque Plus (Amersham Biosciences, Buckinghamshire, United Kingdom), according to routine procedures. PBMCs were washed twice with phosphate-buffered saline (PBS) and re-suspended at 1 × 10^6^ cells/tube for antibody staining.

### Isolation of lamina propria mononuclear cells

LPMCs were isolated from fresh ileum tissue specimens obtained from the subjects, according to a modified version of a protocol reported previously (Hiemstra et al., 2014). Briefly, the surgical mucosal specimens were washed extensively with normal saline and used for isolation of LPMCs within 30 min post resection. The tissue specimens were incubated with Ca^++^/Mg^++^-free Hanks’ balanced salt solution (HBSS, Sigma-Aldrich, St. Louis, United States) containing 2.5% heat-inactivated fetal bovine serum (FBS) and 1 mM dithiothreitol (Sigma-Aldrich) on ice for 10 min. They were then incubated in an HBSS buffer containing 5 mM EDTA, 1 mM dithiothreitol, 2.5% heat-inactivated FBS, and 0.1% (v/v) β-mercaptoethanol (Sigma-Aldrich) at 37°C for 20 min with constant stirring to remove mucus and epithelial cells. Subsequently, the remaining segments were cut into small pieces and digested with 250 μg/ml DNase (Roche, Basel, Switzerland) and 125 μg/ml liberase TM (Roche) in HBSS at 37°C for 30 min with constant stirring. After centrifugation, the cells were re-suspended in RPMI-1640 (Invitrogen, Carlsbad, CA, United States), filtered through a 40-μM cell strainer, and then loaded onto the surface of Ficoll-Paque Plus (GE Healthcare, Little Chalfont, United Kingdom). After another centrifugation at 400 × *g* for 30 min, the LPMCs were recovered from the interface and washed with PBS twice to prepare the cells for flow cytometry.

### Flow cytometric analysis

PBMCs and LPMCs 1 × 10^6^ cells/tube were incubated in an RPMI-1640 medium with 10% FBS and stained in duplicate with anti-human CD3-FITC, anti-human CD4^−^ BV510, anti-human CD8-APC-Cy7, anti-human CD19-PerCP-Cy5.5, anti-human CD161-BV421, anti-human TCRγδ-CF594, anti-human CD8b-PE (BD Biosciences, San Jose, United States), and anti-human TCR Vα7.2-APC (Biolegend, San Diego, California, United States) antibodies at 4°C in the dark for 30 min. After washing, the stained cells were characterized using FACS Aria II (BD Sciences, San Jose, California, United States) and analyzed using FlowJo 7.6.4 software (TreeStar).

### Cytometric bead array analysis of the level of serum cytokines

The concentration of serum TNF-α, IFN-γ, IL-17A, IL-2, IL-4, IL-6, and IL-10 were determined using CBA, according to the manufacturer’s protocol (BD Biosciences). Briefly, individual serum samples (30 µl each) were incubated in duplicate with different types of antibody microbeads for 3 h. After washing, the concentration of the serum cytokines was quantified by flow cytometry on a FACSAria II using the Cell Quest Pro and CBA software (BD Biosciences). The limits for the detection of each cytokine were 2.6 pg/ml (IL-2), 4.9 pg/ml (IL-4), 2.4 pg/ml (IL-6), 4.5 pg/ml (IL-10), 18.9 pg/ml (IL-17A), 3.8 pg/ml (TNF-α), and 3.7 pg/ml (IFN-γ).

### Statistical analysis

The data obtained were expressed as mean, median, and range. The differences between groups were analyzed by the Mann–Whitney U non-parametric test and the relationships between variables were evaluated using the Spearman’s rank correlation test. The difference between pre-treatment and post-treatment patients was analyzed using the Wilcoxon test. All the statistical analyses were performed using the SPSS 19.0 statistical software package (SPSS Inc., Chicago, IL, United States). A two-tailed *p* value <0.05 was considered statistically significant.

## Results

### Patient characteristics

There were no significant differences between the distribution of gender, gestational age, and birth weights, or in the number of WBC and lymphocytes in the peripheral blood samples of NEC patients and the control subjects (Table 1). However, the concentration of serum CRP in the NEC group was found to be significantly higher than that in control subjects. Hence, a high level of inflammation was associated with NEC.

### Alteration in the frequency of circulating CD161 + CD8 + T cells and mucosal-associated invariant T cells in NEC patients

**FIGURE 1:**
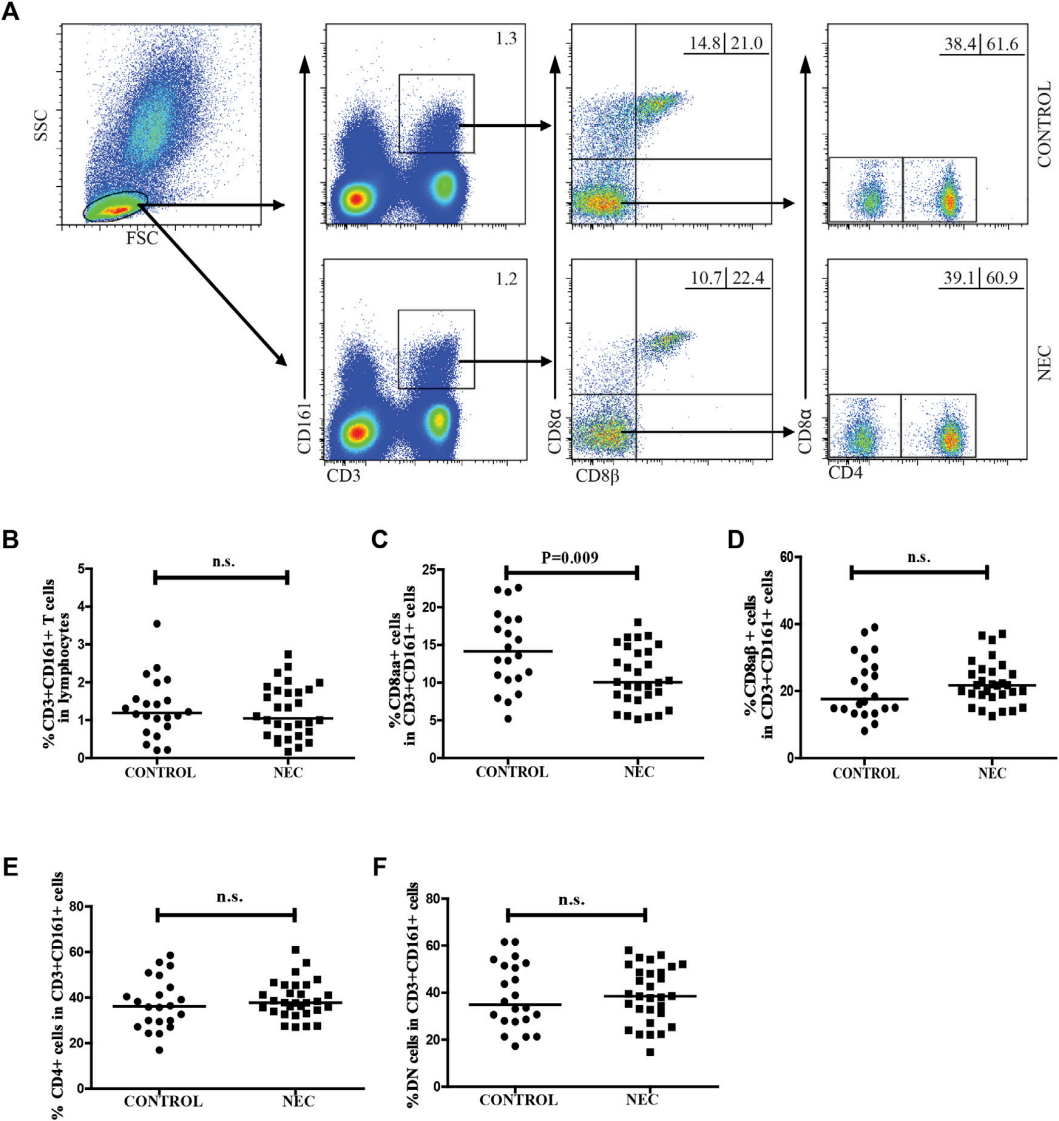
Flow cytometry for analyzing the frequency of circulating CD3^+^ CD161^+^ T cells in patients with NEC. Peripheral blood mononuclear cells (PBMCs) from 30 NEC patients and 22 controls were stained with fluorescent anti-CD3, anti-CD161, anti-CD8α, anti-CD8β, and anti-CD4 antibodies. The cells were gated initially on living lymphocytes, and on CD3^+^ CD161^+^ T cells, and then CD3^+^ CD161^+^ CD8αβ- cells. The percentage of CD3^+^ CD161^+^, CD3^+^ CD161^+^ CD8αα^+^, CD3^+^ CD161^+^ CD8αβ^+^, CD3^+^ CD161^+^ CD4^+^, and CD3^+^ CD161^+^ DN T cells in the subjects were analyzed by flow cytometry. **(A)** Flow cytometric analysis. **(B–F)** Quantitative analysis. Data are representative dot plots or expressed as the mean percentage of cells in individual subjects. The difference between the two groups was analyzed by Mann–Whitney U nonparametric test. The horizontal lines represent the median values.

We used flow cytometry to characterize the frequency of different subsets of circulating CD3 + CD161 + CD8 + T cells in patients with NEC and the controls. As shown in Figure 1, there was no significant difference in the frequency of CD3 + CD161 + cells in total lymphocytes. In addition, there were no differences in the percentage of CD3 + CD161 + CD8αβ +, CD3 + CD161 + CD4 +, and the DN subsets of cells in total CD3 + CD161 + T cells between the two groups. However, the percentage of circulating CD3 + CD161 + CD8αα + T cells in total CD3 + CD161 + T cells in the NEC patients was significantly lower than that in the controls (*p* = 0.009).

**FIGURE 2:**
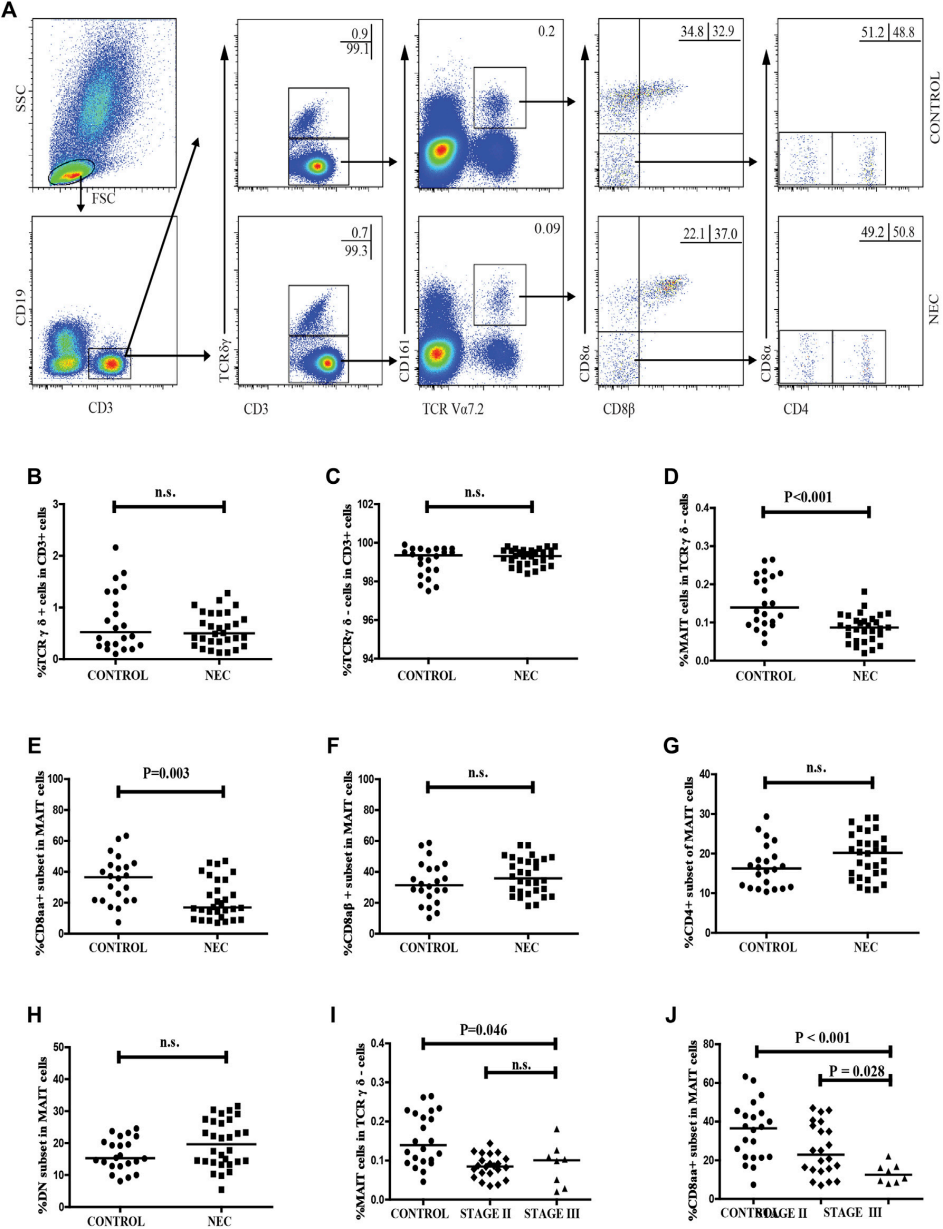
Flow cytometric analysis of the frequency of circulating MAIT cells in NEC patients. Peripheral blood mononuclear cells (PBMCs) from NEC patients and controls were stained with fluorescent anti-CD3, anti-CD19, anti-CD161, anti-TCRγδ, anti-TCR Vα7.2, anti-CD8α, anti-CD8β, and anti-CD4 antibodies. The cells were gated sequentially on living lymphocytes, CD19^−^CD3 + T cells, CD3 + TCRγδ-cells, TCR Vα7.2 + CD161 +, and then TCR Vα7.2 + CD161 + CD8αβ- MAIT cells. The percentage of CD3 + TCRγδ-, CD3 + TCRγδ +, CD3 + TCRγδ-Vα7.2 + CD161 + MAIT, CD8αα +, CD8αβ +, CD4 + and DN MAIT cells was analyzed by flow cytometry. **(A)** Flow cytometry analysis. **(B–H)** Quantitative analysis. **(I–J)** Stratification analysis of the percentage of circulating MAIT cells in NEC patients. Data shown are representative dot plots or expressed as the mean percentage of cells in individual subjects. The difference between these two groups was analyzed using the Mann–Whitney U nonparametric test. The horizontal lines represent the median values.

We estimated the percentage of circulating CD3 + TCRγδ-Vα7.2 + CD161 + MAIT cells in total CD3 + TCRγδ- T cells in NEC patients and control subjects using flow cytometry (Figure 2A). We did not find a significant difference between the frequency of circulating CD3 + TCRγδ + T cells or CD3 + TCRγδ- T cells in CD3 + T cells in the NEC patients and the control subjects (Figures 2B,C). The percentage of circulating CD3 + TCRγδ-Vα7.2 + CD161 + MAIT cells in total CD3 + TCRγδ- T cells in NEC patients was found to be significantly lower than that in the control subjects (*p* < 0.001, Figure 2D). Further analysis of the different subsets of MAIT cells showed that the percentage of CD8αα + MAIT cells in total MAIT cells were significantly lower in patients with NEC than in control subjects (*p* = 0.003, Figure 2E). However, there was no significant difference between the percentage of CD8αβ+, CD4+, and DN MAIT cells in total MAIT cells of the two groups (Figures 2F–H). In addition, we found that when the 30 NEC patients were classified based on the severity of NEC (stage II/III, *n* = 22/8), there was no significant difference between the percentage of circulating MAIT cells in CD3 + TCRγδ- T cells in patients with stage II and stage III NEC (Figure 2I). In contrast, the percentage of CD8αα + MAIT cells in total MAIT cells in NEC patients at stage III was significantly lower than that in stage II NEC patients (*p* = 0.028, Figure 2J). We also found no significant difference between the percentage of other subsets of MAIT cells in patients with stage II and stage III NEC (Supplementary Figures S1A–C).

### Changes in the frequency of CD161 + CD8 + T cells and MAIT cells in the intestinal tissues of NEC patients and control subjects

**FIGURE 3:**
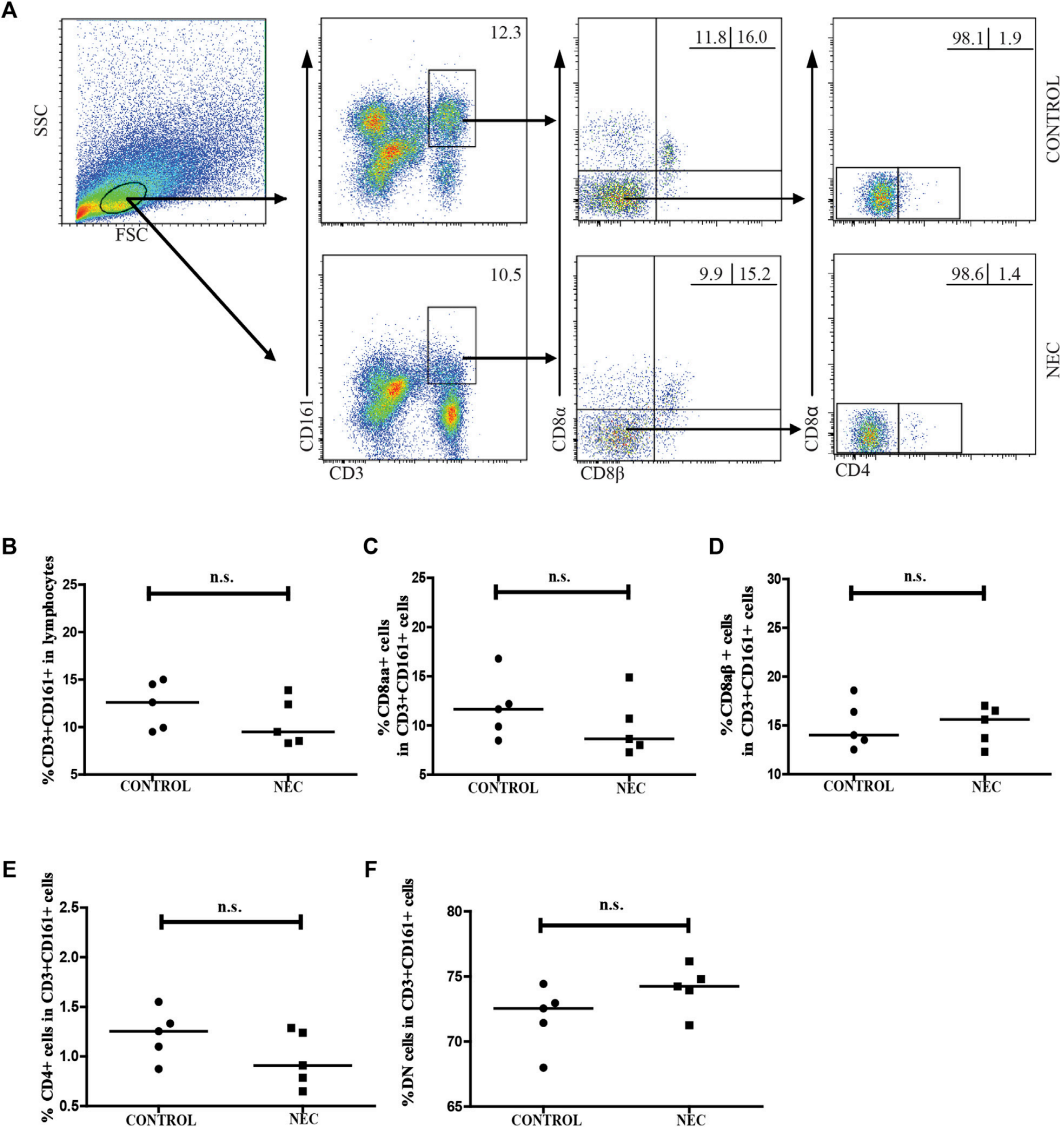
Flow cytometric analysis of the frequency of CD161 + CD3 + cells in the intestinal tissues of NEC patients. Lamina propria mononuclear cells (LPMCs) from five NEC patients and five non-NEC controls were stained with fluorescent anti-CD3, anti-CD161, anti-CD8α, anti-CD8β, and anti-CD4 antibodies. The cells were gated sequentially on living lymphocytes, and on CD3 + CD161 + T cells, and then CD3 + CD161 + CD8αβ- cells. The percentage of CD3 + CD161 + T cells in lymphocytes and of CD3 + CD161 + CD8αα +, CD3 + CD161 + CD8αβ +, CD3 + CD161 + CD4 +, and CD3 + CD161 + DN T cells in total CD3 + CD161 + T cells were analyzed by flow cytometry. **(A)** Flow cytometry analysis. **(B–F)** Quantitative analysis. Data shown are representative dot plots or expressed as the mean percentage of cells in individual subjects. The difference between the two groups was analyzed using the Mann–Whitney U nonparametric test. The horizontal lines represent the median values.

We analyzed the distribution of CD3 + CD161 + cells and MAIT cells in the intestinal tissues of NEC patients and control subjects. LPMCs were isolated from five infants with advanced NEC and five non-NEC patients who underwent surgery for a non-inflammatory disease (3 cases with congenital intestinal bowel obstruction and 2 cases with severe congenital intestinal malrotation). As shown in Figure 3, there was no significant difference between the frequency of CD3 + CD161 + T cells in total lymphocytes, or the percentage of CD3^+^ CD161^+^ CD8αα^+^, CD3^+^ CD161^+^ CD8αβ^+^, CD3^+^ CD161^+^ CD4^+^, and DN T cells in total CD3^+^ CD161^+^ T cells in the intestinal tissues of NEC patients and control subjects.

**FIGURE 4:**
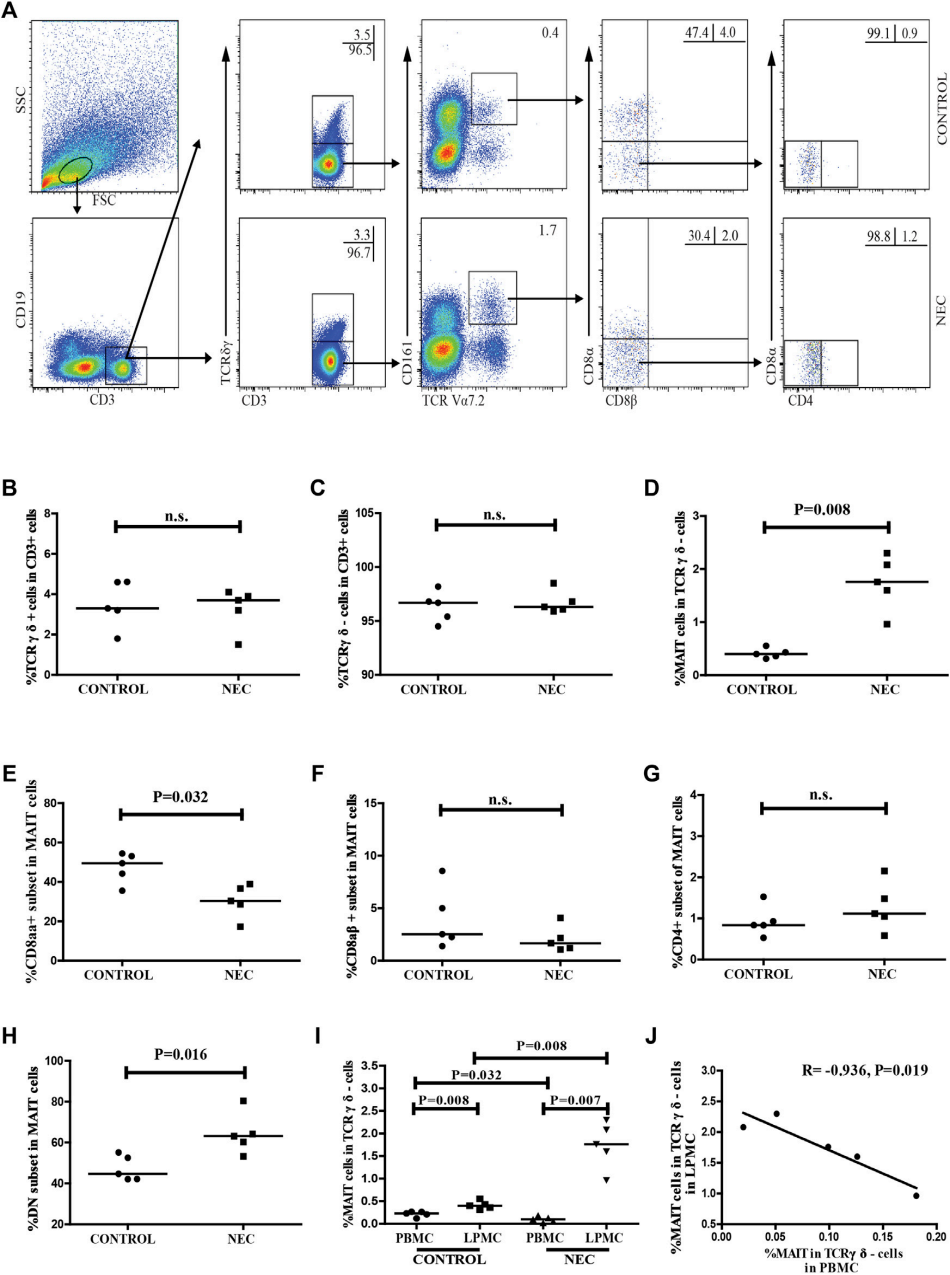
Flow cytometric analysis of the frequency of MAIT cells in the intestinal tissues of NEC patients. Lamina propria mononuclear cells (LPMCs) from NEC patients and non-NEC controls were stained with fluorescent anti-CD3, anti-CD19, anti-CD161, anti-TCRγδ, anti-TCR Vα7.2, anti-CD8α, anti-CD8β, and anti-CD4 antibodies. The cells were gated sequentially on living lymphocytes, CD3 + CD19^−^ T cells, CD3 + TCRγδ- cells, and TCR Vα7.2 + CD161 + MAIT cells, and then Vα7.2 + CD161 + CD8αβ- MAIT cells. The percentage of CD3 + TCRγδ-, CD3 + TCRγδ +, CD3 + TCRγδ-TCRVα7.2 + CD161 + MAIT, CD8αα +, CD8αβ +, and CD4 + and DN MAIT cells were analyzed by flow cytometry. **(A)** Flow cytometry analysis. **(B–H)** Quantitative analysis of cells in the LPMCs from the control subjects and NEC patients. **(I)** Quantitative analysis of the percentage of circulating and intestinal MAIT cells in the control and NEC groups. **(J)** Correlation between the percentage of MAIT cells in PBMCs and LPMCs of NEC patients. Data shown are representative dot plots or expressed as the mean percentage of cells in individual subjects. The difference between the two groups was analyzed by the Mann–Whitney U nonparametric test and their correlation was also analyzed. The horizontal lines represent the median values.

We also used flow cytometry to analyze the frequency of the different subsets of MAIT cells in LPMCs (Figure 4A). We found no significant difference between the percentage of CD3 + TCRγδ + T cells or CD3 + TCRγδ- T cells in NEC patients and control subjects (Figures 4B,C). Compared with the control subjects, the frequency of MAIT cells in NEC patients was significantly higher (*p* = 0.008, Figure 4D). However, the percentage of CD8αα + MAIT cells in total MAIT cells was significantly lower in the NEC patients (*p* = 0.032, Figure 4E). The frequency of DN MAIT cells in total MAIT cells was significantly higher in the NEC patients (*p* = 0.016, Figure 4H). There was no significant difference between the percentage of CD8αβ^+^ or CD4^+^ MAIT cells in the two groups (Figures 4F,G). Interestingly, the percentage of MAIT cells in CD3 + TCRγδ- LPMCs was found to be significantly higher than that of circulating MAIT cells in CD3 + TCRγδ- T cells in both control and NEC groups (*p* = 0.008 and *p* = 0.007, respectively; Figure 4I). Additionally, the percentage of circulating MAIT cells in NEC patients was significantly lower than that in control subjects (*p* = 0.032, Figure 4I). In contrast, the frequency of MAIT cells in the LPMCs of NEC patients was significantly higher than that in the LPMCs of the control group (*p* = 0.008, Figure 4I), and the percentage of MAIT cells in the peripheral blood was inversely correlated with that in the intestinal tissues of NEC patients (R = −0.936, *p* = 0.019, Figure 4J). Thus, alterations in the frequency of MAIT cells were likely associated with the development of NEC.

### Variable serum levels of inflammatory cytokines and correlation analysis between MAIT cells, and CD8αα + MAIT cells in NEC patients and control subjects

**FIGURE 5:**
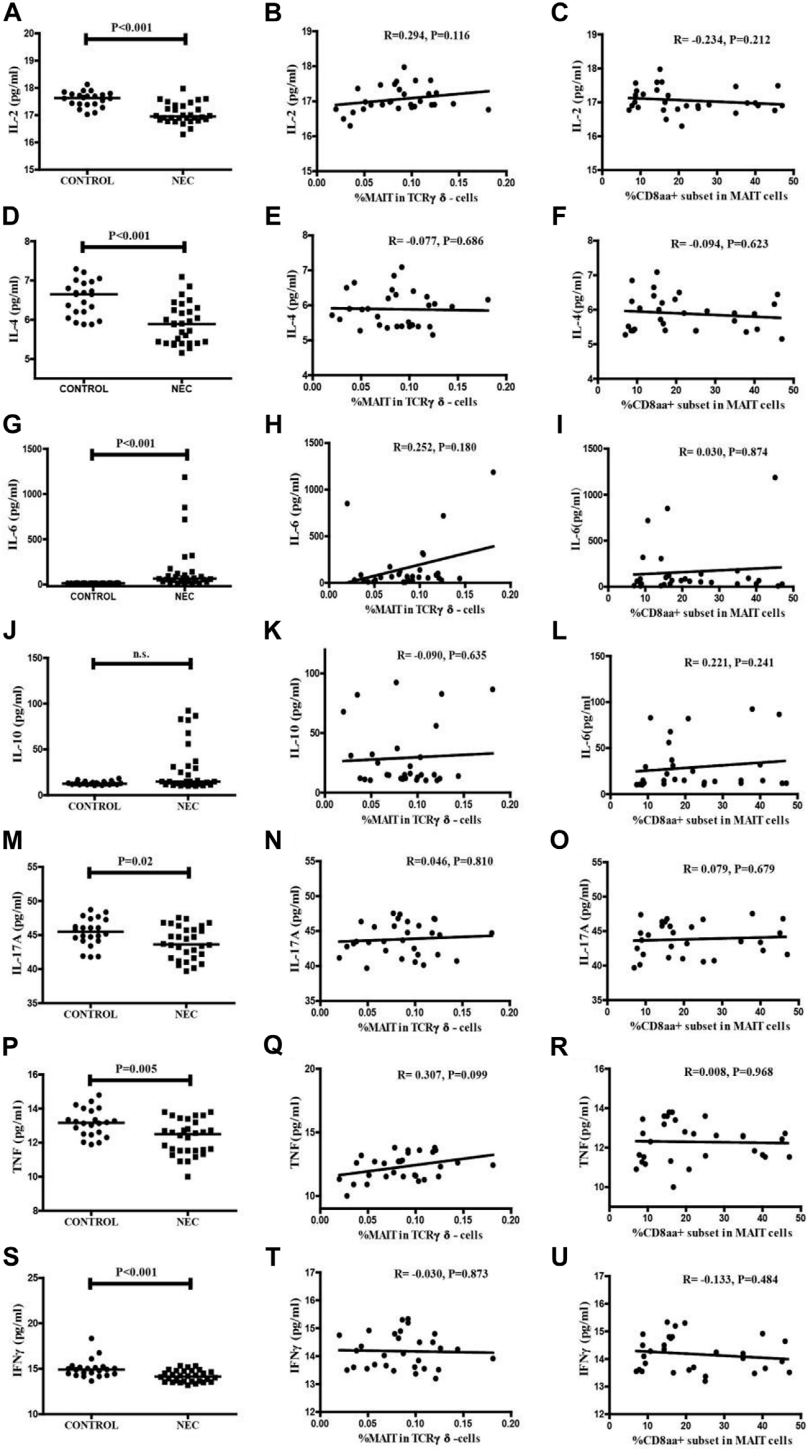
Levels of serum cytokines in the NEC patients and controls. The levels of serum IL-2, IL-4, IL-6, IL-10, IL-17, TNF-α, and IFN-γ in individual subjects were determined by CBA. **(A,D,G,J,M,P,S)** The serum levels of IL-2, IL-4, IL-6, IL-10, IL-17, TNF-α, and IFN-γ in the control subjects and NEC patients. Data shown are expressed as the mean values in individual subjects. The difference between the two groups was analyzed by the Mann–Whitney U nonparametric test. The horizontal lines represent the median values. Correlations among the percentages of circulating MAIT cells and the levels of serum inflammation cytokines in NEC patients. Potential correlations among the percentages of circulating MAIT cells and the levels of serum cytokines were analyzed by the Spearman correlation tests. **(B,E,H,K,N,Q,T)** The percentages of circulating MAIT cells showed no significant correlation with the levels of serum cytokines in NEC patients. Correlations among the percentages of circulating CD8αα + MAIT cells and the levels of serum inflammation cytokines in NEC patients. Potential correlations among the percentages of circulating CD8αα + MAIT cells and the levels of serum cytokines were analyzed by the Spearman correlation tests. **(C,F,I,L,O,R,U)** The percentages of circulating CD8αα + MAIT cells showed no significant correlations with the levels of serum cytokines in NEC patients.

The level of serum cytokines in NEC patients and controls were analyzed using CBA (Figures 5A,D,M,P,S). The levels of serum IL-2, IL-4, IL-17A, TNFα, and IFNγ in the NEC patients were significantly lower than those in the controls (*p* < 0.001, *p* < 0.001, *p* = 0.02, *p* = 0.005, *p* < 0.001, and respectively). In contrast, the level of serum IL-6 in the NEC patients was significantly higher than that in the controls ((Figure 5G, *p* < 0.001). The difference in the level of serum IL-10 in the two groups was not significant (Figure 5J).

In addition, we investigated the association between these changes in the frequency of circulating MAIT cells and CDαα + MAIT cells and the disease activity. We analyzed the correlations between the levels of serum cytokines and the percentage of MAIT cells (Figures 5B,E,H,K,N,Q,T) and CD8αα + MAIT cells (Figures 5C,F,I,L,O,R,U), and found no significant correlation. We further found there was not significant correlation with the association between the level of CRP and the percentage of MAIT cells and CD8αα + MAIT cells (Supplementary Figure S2). This result could be explained by the fact that cytokines are secreted by several types of immune cells, or by the lack of known clinical values of these measures in patients with NEC.

### Clinical parameters and frequency of MAIT cells and CD8αα + MAIT cells in NEC patients after treatment

**FIGURE 6:**
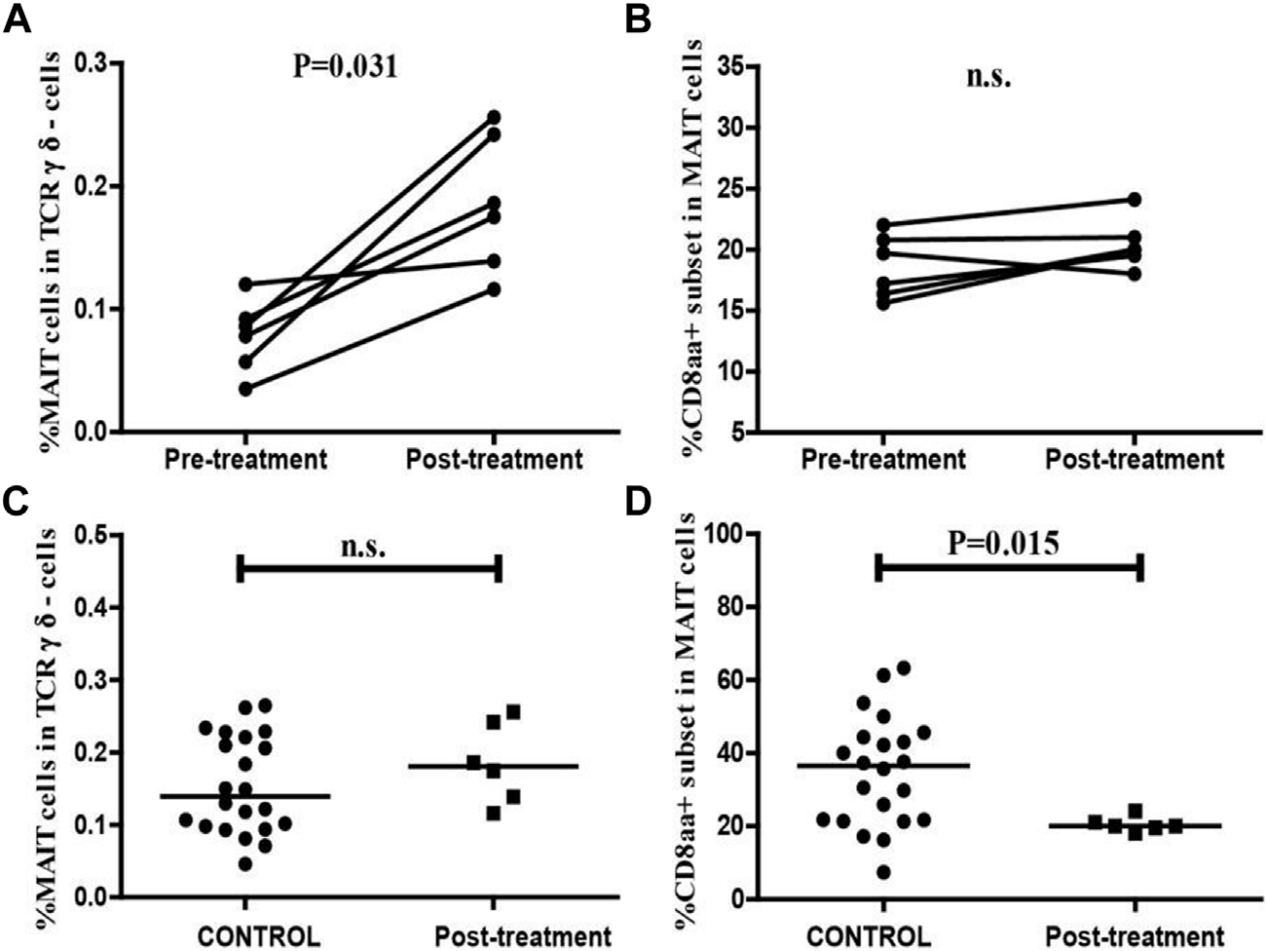
Altered frequencies of MAIT cells and CD8αα + MAIT cells in NEC patients after treatment. The percentage of MAIT cells and CD8αα + MAIT cells were analyzed in NEC patients (*n* = 6) before and after treatment using the Wilcoxon test. The difference between the percentage of circulating MAIT cells and CD8αα + MAIT cells in the control and after-treatment groups was analyzed by the Mann–Whitney U nonparametric test. **(A,B)** The percentage of circulating MAIT cells and CD8αα + MAIT cells in individual patients before and after treatment. **(C,D)** Quantitative analysis of the percentage of circulating MAIT cells and CD8αα + MAIT cells in the controls and NEC patients after treatment. Horizontal lines represent the median values.

To better understand the association between MAIT cells and NEC, we assessed the values of clinical parameters and the percentage of circulating MAIT cells and CD8αα + MAIT cells in six patients before and 2–3 weeks after treatment. The remaining 24 patients failed to follow-up or had subsequent infections. After treatment, none of the six patients displayed any signs of NEC or other infectious diseases, based on clinical presentation and radiological examination, and their serum CRP levels were found to be dramatically decreased. There was no significant difference between the WBC and lymphocyte counts before and after treatment (Table 2). We also found that the percentage of circulating MAIT cells was significantly higher in patients after treatment than in those before treatment (*p* = 0.031, Figure 6A), and were similar to that in the controls (Figure 6C). However, there was no significant difference between the percentage of circulating CD8αα^+^ MAIT cells in patients after treatment and those before treatment (Figure 6B). In addition, the percentage of circulating CD8αα^+^ MAIT cells in the patients after treatment remained significantly lower than that in the controls (*p* = 0.015, Figure 6D).

**TABLE 2:** Demographic and clinical characteristics of follow-up NEC patients.

Parameters	Pre-treatment	Post-treatment
Male/Female (n)	2/4	2/4
White blood cells (10^9^/L)	10.8 (4.5–18.2)	10.6 (6.6–15.0)
Lymphocytes (10^9^/L)	3.9 (2.9–5.6)	3.8 (2.3–4.6)
CRP (mg/L)	6.3 (1.2–11.07)	2.1 (0.6–3.7)*

Data are expressed as the median (range or case number). *p < 0.05 vs. the control.

## Discussion

The immune system plays a crucial role in the pathogenesis of NEC. MAIT cells are a subset of unconventional, innate-like T cells that primarily accumulate at sites where exposure to microbes is ubiquitous (Napier et al., 2015) and secretes pro-inflammation cytokines and cytotoxic mediators in response to pathogens and infected cells (Gold et al., 2010; Le Bourhis et al., 2010). MAIT cells regulate immune responses in inflammatory diseases such as the inflammatory bowel disease (Serriari et al., 2014; Booth et al., 2015; Barathan et al., 2016). However, the role of MAIT cells in the pathology of NEC in preterm infants remains unknown.

In this study, we examined the frequency, distribution, and clinical relevance of different subsets of MAIT cells in peripheral blood and intestinal tissues of NEC patients and control subjects. We found that the frequency of MAIT cells was significantly decreased in the peripheral blood, but significantly increased in the inflamed intestinal tissues, of NEC patients compared with control subjects. In both peripheral blood and intestinal tissues, the frequency of CD8αα + MAIT cells was significantly lower in NEC patients than in the control subjects. To the best of our knowledge, this is the first study to describe the changes in the frequency and distribution of MAIT cells during the development of NEC.

To determine the distribution of MAIT cells in NEC patients, we analyzed their frequency in peripheral blood and intestinal tissues of NEC patients and control subjects. We found that MAIT cells were more frequently observed in the intestinal tissues than in the peripheral blood, in both control and NEC patients. A potential explanation of this is that the gastrointestinal tract of the newborn is exposed to a plethora of microbes immediately after birth. MAIT cells might preferably reside in the intestinal mucosa to regulate the immunity and might be activated by non-polymorphic major histocompatibility (MHC) class I-related molecule (MR1)-restricted vitamin derivatives, which originate from the bacterial riboflavin (vitamin B2) biosynthetic pathway (Kjer-Nielsen et al., 2012). Compared with the control subjects, the frequency of MAIT cells was significantly low in the peripheral blood of NEC patients. In contrast, it was significantly higher in the intestinal tissues of the NEC patients than the control. Interestingly, the percentage of MAIT cells in the peripheral blood was inversely correlated with that in the intestinal tissues of NEC patients. These data suggested that circulating MAIT cells might migrate to and accumulate in the intestinal tissues. Our findings lent support to previous observations that a reduction in the frequency of circulating MAIT cells was associated with their infiltration into inflammatory tissues (Serriari et al., 2014; Willing et al., 2014; Ling et al., 2016). These data indicated that the MAIT cells accumulated in the inflamed intestinal tissues during NEC development.

It was previously demonstrated that CD8 + MAIT cells had more potent inflammatory functions and higher cytotoxicity than DN MAIT cells (Brozova et al., 2016). CD8 + MAIT cells have been divided into CD8αα and CD8αβ subsets (Walker et al., 2012). Several studies have suggested that CD8αα + MAIT cells are more matured than CD8αβ + MAIT cells in human fetus and animal models (Konno et al., 2002; Poussier et al., 2002; Walker et al., 2012; Leeansyah et al., 2014). In this study, we found that the frequency of CD8αα + MAIT cells was significantly reduced in both peripheral blood and intestinal tissues of NEC patients, compared with the control subjects. Furthermore, a reduction in circulating CD8αα + MAIT cells was associated with the severity of NEC. These data were consistent with a previous report, which suggested that the frequency of CD8αα + innate-type lymphocytes was reduced in the intestinal epithelium of NEC patients (Van Kaer et al., 2014). This result suggested that the decreased frequency of CD8αα + MAIT cells might contribute to the pathogenesis of NEC in humans with unknown mechanism.

CD161 + CD8 + T cells are known to overlap Tc17 and MAIT cell populations, which are derived from a pool of pre-committed CD161^high^ T cells (Billerbeck et al., 2010; Walker et al., 2012). CD161 + CD8 + T cells are found at potential sites of pathogen entry within epithelia, and play an important role in host defense, particularly in lamina propria (Fergusson et al., 2016; Rout, 2016). In this study, we found that the frequency of CD3 + CD161 + CD8αα + T cells was significantly reduced in the peripheral blood of preterm infants with NEC, consistent with the change in the frequency of circulating CD8aa + MAIT cells in NEC patients. However, there was no significant difference between the frequency of CD161 + CD8 + T cells in the intestinal tissues of NEC patients and controls.

We also analyzed the serum level of inflammatory cytokines in NEC and control subjects, and detected significantly lower levels of serum IL-2, IL-4, TNFα, IFNγ, and IL-17A, and a higher level of IL-6, in NEC patients. However, there was no correlation between the levels of serum cytokines and the percentage of CD8αα + and MAIT cells. The reduced levels of serum pro-inflammatory cytokines were consistent with a previous report (Benkoe et al., 2013). This reduction might be explained by the systemic deterioration, particularly of immunocompetent cells in patients with NEC. It would be interesting to further investigate the lower levels of pro-inflammatory cytokines in patients with NEC.

We also found no significant correlation between the level of serum CPR and the percentage of MAIT and CD8αα + MAIT cells in NEC patients. This might be because CRP level is not a specific clinical value. Currently, no specific clinical values or biomarkers are available to diagnose NEC. This limitation needs to be eliminated to further analyze pathology of this disease.

After medical management with supportive care, antibiotic therapy (Solomkin et al., 2010), and close laboratory and radiological examinations, we found that the frequency of circulating MAIT cells in NEC patients significantly increased and reached a level similar to that in control subjects. This suggested that MAIT cells might be active participants in the pathogenesis of NEC. However, there was no significant difference between the frequency of circulating CD8αα + MAIT cells before and after treatment, and the percentage of circulating CD8αα + MAIT cells in NEC patients after treatment remained lower than that in the control subjects. A possible explanation is that there are genetic factors that affect CD8αα expression or evolution of CD8αα + cells, which would explain the predisposition of some premature infants to NEC. Similar results were reported in a study conducted in obese patients, MAIT cells were more abundant in adipose tissue than in the blood and exhibited a striking IL-17 profile. Bariatric surgery in obese patients also increased circulating MAIT cell frequency at 3 months after surgery (Magalhaes et al., 2015). Overall, inflammation may have a major impact on circulating and peripheral tissue MAIT cell frequency and function: circulating MAIT cell frequency was profoundly decreased. MAIT cell abnormalities could be markedly attenuated after treatment. The results paves the way for deeper investigation of the mechanisms responsible for the redistribution of MAIT cells.

It should be noted that MAIT cells also play a role in sterile inflammation such as autoimmune disease (AID) when considering the role of CD8αα + MAIT in NEC. An important feature of MAIT cells is that they can be stimulated within the cytokine milieu without specific TCR stimulation. Hence, human and murine MAIT cells can produce IL-17, IFN-γ and TNF-α in response to IL-18 and IL-23 stimulation, and amplify pro-inflammatory signals non-specifically, enhancing inflammation in sterile inflammation conditions (Hinks, 2016). However, both protective and pathogenic roles of MAIT cells during AID were observed (Howson et al., 2015). So how coordinate regulation of the contrasting role of MAIT cells in AID is mediated?

MAIT cells has low-affinity TCR and are coreceptor dependent for their activation and initial selection (Walker et al., 2013). CD8αβ, but not CD8αα, functions as a coreceptor which physically interacts with TCR-CD3, hence substantially enhances raft association of TCR-CD3 and the antigen sensitivity. In contrast to CD8αβ, CD8αα functions as a TCR repressor and co-expression of CD8αα effectively decreases antigen sensitivity of TCRs and markedly diminishes or completely abolishes T cells activation (Cawthon et al., 2001). Further, because CD8αα can be transiently induced on chronically activated CD8αβ + T cells (Madakamutil et al., 2004) or constitutively expressed on intraepithelial lymphocytes (IEL) of the small intestine (Cheroutre, 2004), it could either temporarily lower the functional avidity and attenuate an ongoing immune response or permanently increase the threshold required for restimulation of pathogen-experienced effector and memory T cells that reside within the antigen-rich environment of the gut (Cheroutre and Lambolez, 2008). Thus intestinal T cells upregulate CD8αα to assist in the adaptation to the microenvironment of the intestine and keep in immunologically quiescent state in the locales (Ma et al., 2019).

Additionally, in healthy organism CD8αα is most highly expressed by MAIT cells, with CD8αα expression highly restricted to the TCR Vα7.2 + cells of this subset, which is consistent with our observation in intestine of control group (Walker et al., 2013). It may suggest a function for CD8αα + MAIT cells in gut immune tolerance. While in NEC premature, decreased proportion of CD8αα + MAIT cells in intestine may depict an immune tolerance hypothesis for pathogenesis of NEC. An immune tolerance related mechanism may be involved in preterm infants NEC pathogenesis, but more functional studies are needed to verify this hypothesis.

Finally we recognized that our studies had several limitations, including a small sample size and the lack of functional studies of MAIT cells in the pathogenesis of NEC. Besides, another limitation of this study was that we did not use dead cell markers. Although a significant difference was found in the frequencies of circulating MAIT cells, CD8αα + MAIT cells, and CD3 + CD161 + CD8αα + T cells between the control and NEC groups, conclusions should be drawn carefully as the groups overlap in Figures 1, 2. Further validation of these findings and hypothesis in a bigger population through mechanism studies would be required to elucidate the role of MAIT cells in the pathogenesis of NEC.

## Conclusion

In summary, our data indicated a reduction in the frequency of MAIT cells in peripheral blood, but an increase in the percentage of MAIT cells in the intestinal tissues of NEC patients. Additionally, the percentage of CD8αα + MAIT cells was found to be decreased in both peripheral blood and intestinal tissues of N EC patients. The percentage of circulating CD8αα + MAIT cells was also associated with the severity of NEC. Hence, our findings may provide new insights into the role of different subsets of MAIT cells in the pathogenesis of NEC. Further investigations should be performed to examine the function of different subsets of MAIT cells in the development of NEC.

## Data Availability

The original contributions presented in the study are included in the article/Supplementary Material, further inquiries can be directed to the corresponding author.
