# Supplementary material for: Peripheral and intestinal mucosal-associated invariant T cells in premature infants with necrotizing enterocolitis
Source: Front Pharmacol. 2022 Sep 14;13:1008080. doi: 10.3389/fphar.2022.1008080 (PMC9515899; doi:10.3389/fphar.2022.1008080)
Supplement: Supplementary file 1 [file Presentation1.pdf]

Supporting Information

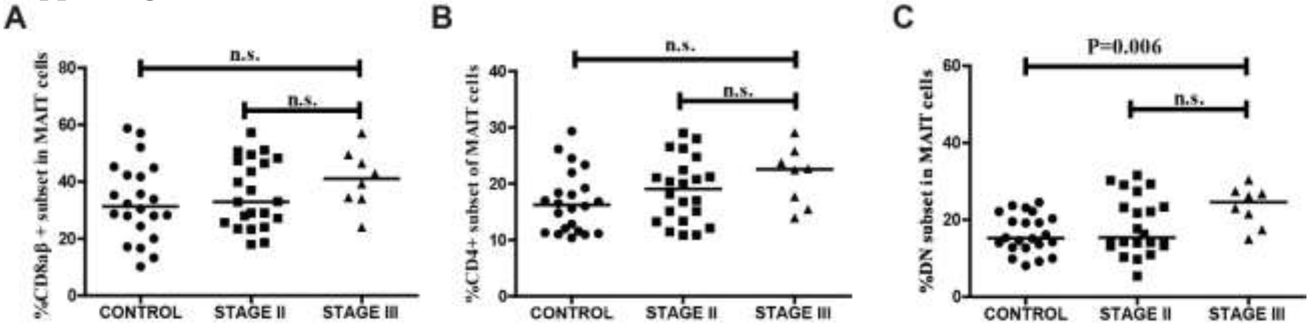

Figure S1. Stratification analysis of the percentages of circulating MAIT cells in NEC patients. The patients were stratified, according to the disease stages (stage II, n=22 and stage III, n=8), and the percentage of different subsets of MAIT cells were analyzed in individual patients and controls. **(A)** Quantitative analysis of the percentages of $CD8\alpha\beta^+$ MAIT in total MAIT cells. **(B)** Quantitative analysis of the percentage of $CD4^+$ MAIT in total MAIT cells. **(C)** Quantitative analysis of the percentage of DN MAIT in total MAIT cells.

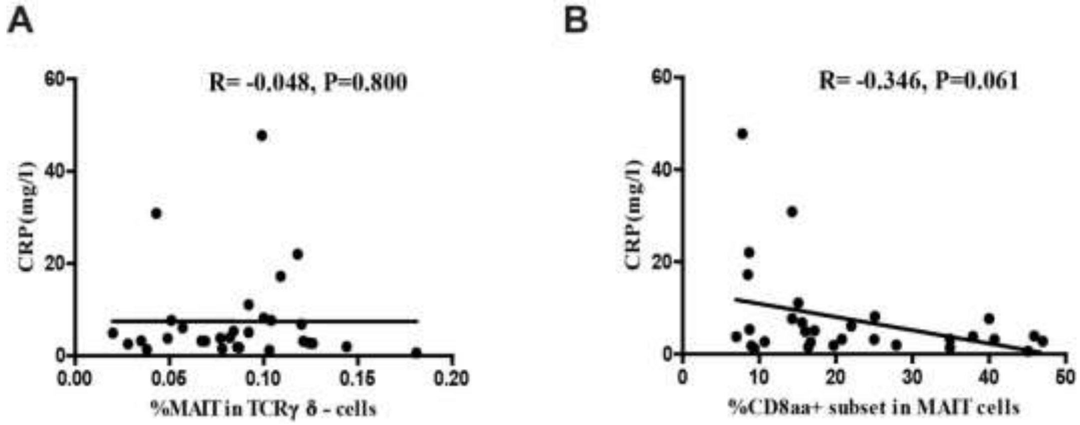

Figure S2. Correlations among the percentages of circulating MAIT cells, $CD8\alpha\alpha^+$ MAIT cells and the levels of serum C-reactive protein (CRP) in NEC patients. Potential correlations among the percentages of circulating MAIT cells, $CD8\alpha\alpha^+$ MAIT cells and the levels of serum CRP were analyzed by the Spearman correlation tests. **(A-B)** The percentages of circulating MAIT cells, $CD8\alpha\alpha^+$ MAIT cells showed no significant correlations with the levels of serum CRP in NEC patients.
